# Cavovarus deformity in Charcot-Marie-Tooth disease: is there a hindfoot equinus deformity that needs treatment?

**DOI:** 10.1186/s13047-015-0121-6

**Published:** 2015-11-26

**Authors:** Nicholas A. Beckmann, Sebastian I. Wolf, Daniel Heitzmann, Annika Wallroth, Sebastian Müller, Thomas Dreher

**Affiliations:** Clinic for Orthopaedics and Trauma Surgery, Heidelberg University Hospital, Schlierbacher Landstrasse 200A, 69118 Heidelberg, Germany; Heidelberg Motion Lab, Clinic for Orthopaedics and Trauma Surgery, Heidelberg University Hospital, Schlierbacher Landstrasse 200A, 69118 Heidelberg, Germany

## Abstract

**Background:**

Charcot-Marie-Tooth disease (CMT), one of the most common hereditary neurologic disorders, often results in debilitating cavovarus foot deformities. The deformities are still not fully understood, and the treatment recommendations are consequently heterogeneous, often including calf muscle or Achilles tendon lengthening.

**Methods:**

We examined 40 patients (80 feet) with CMT and bilateral cavovarus deformities (19 men and 21 women, mean age 33.6 ± 14.6 years) and the feet of a healthy control population of 13 individuals (7 men and 6 women, mean age 43.9 ± 10.8 years). In all cases 3D instrumented gait analysis results with both conventional Plug-in-Gait analysis and the Heidelberg Foot Measurement Method (HFMM) were used to determine the sagittal plane kinematics, dorsi-plantar flexion (DPF), tibio-talar dorsiflexion (TTDF), and medial arch angle (MAA), and the results of patients and the control group were compared using the 2 methods. Decreased and increased dorsiflexion using TTDF was defined as 1 standard deviation below or above the mean of the control. Comparisons were done using descriptive statistics, the Pearson correlation coefficient and ANOVA.

**Results:**

The TTDF was found to be decreased in 18 of the 80 feet examined (22.5 %), normal in 31 feet (38.75 %), and increased in 31 feet (38.75 %). The Pearson coefficient showed a positive correlation with *R* = 0.765, *p* < 0.001 between decreased TTDF values found by HFMM and decreased DPF values found with conventional Plug-in-Gait analysis, but a very weak correlation in patients with normal TTDF (*R* = -0.118) and increased TTDF (*R* = 0.078). Also, in patients with decreased TTDF values, there was a weak to moderate correlation with the MAA (*R* = 0.335), but no correlation between the MAA and DPF (*R* = 0.023).

**Conclusions:**

The HFMM, unlike the conventional Plug-in-Gait analysis, distinguishes between the segments of the foot in foot deformities and facilitates evaluation of the hindfoot equinus component in patients with CMT and cavovarus deformity. Although there is a significant correlation between decreased TTDF with HFMM and decreased DPF with conventional Plug-in-Gait analysis, this correlation was not seen in patients with normal or increased TTDF values. Conventional Plug-in-Gait analysis alone does not indicate if an increased plantar flexion deformity is the result of either a cavus deformity or hindfoot equinus deformity, which limits its usefulness in assisting in treatment decision making.

## Background

Charcot-Marie-Tooth (CMT) disorder, also known as hereditary motor and sensory neuropathy (HMSN) [1], or peroneal muscular atrophy [2], is the most frequently inherited neuropathy [1, 3, 4]. It is genetically heterogeneous, involving over 80 mutated genes with variable patterns of inheritance [5]. The majority of cases belong to the CMT1 group, with a demyelinating pattern of denervation. Subgroup CMT1A comprises 70 % of all CMT patients, and is associated with a defect on chromosome 17 that codes for peripheral myelin protein [6]. CMT2 cases show an axonal pattern of denervation [6].

Patients with CMT show progressive muscular atrophy and weakness leading to deformity of the feet and less commonly of the hands and progressing proximally. Patients display varying deformities and rates of progression depending on their genetic and phenotypic make-up. The disease results in a multiplanar foot deformity, with cavovarus being the most commonly observed deformity. Wines et al. found an overall incidence of cavovarus in 66 % of their series of 104 feet of 52 patients, with CMT1 patients having the highest incidence. They found a lower incidence of cavovarus (36 %) in the less frequently encountered CMT2 group, and a higher incidence of planovalgus (55 %) [7]. The cavovarus deformity is characterized by hindfoot varus, cavus, plantar flexion of the first metatarsal, adducted forefoot and claw toes. It results from a muscle imbalance between a relatively strong peroneus longus muscle and a weak anterior tibial muscle or between a strong posterior tibial muscle and a weak peroneus brevis muscle [2, 8]. Patients with cavovarus deformity, CMT and varying degrees of sensory loss experience muscle weakness, painful foot callosities, abnormal gait and ankle instability [6, 8–11]. The muscle imbalance involving a weak anterior tibial muscle and strong peroneus longus muscle can also cause foot drop and an equinus deformity of the hindfoot [2, 9]. All these symptoms can occur concurrently and with varying severity depending on the degree and the location of neural involvement.

When conservative treatment options have been exhausted, surgical treatment becomes necessary. A standard treatment algorithm is still lacking, and a variety of treatment strategies exist in order to address the different manifestations of the disease [9]. Appropriate treatment addresses the issue of whether the forefoot loading in these patients is solely a result of the cavus and plantar flexed 1^st^ ray, or alternatively, is the result of a coexistent equinus deformity of the hindfoot that requires additional treatment during the cavovarus deformity correction. Common corrective procedures for hindfoot equinus deformity include Achilles tendon or calf muscle lengthening [2, 12, 13]. However, the utilization of these lengthening procedures has been the cause of some concern since in some instances there has been resultant further weakening of the plantar flexor muscles. This has led to attempts to further identify and define the equinus deformity by utilizing conventional 3 dimensional Plug-in-Gait analysis data [6, 14]. However, conventional Plug-in-Gait analysis has proved inadequate for identifying a true equinus deformity of the hindfoot, leading to the use of more elaborate methods of gait analysis, including the Heidelberg Foot Measurement Method (HFMM) [15], that can differentiate motion between foot segments and thus evaluate hind and fore foot motion [16–18].

The goal of this study was to evaluate the true equinus deformity specifically affecting the hindfoot in patients with CMT and cavovarus, utilizing both conventional Plug-in-Gait analysis and the HFMM, and compare the two analysis methods in their ability to identify equinus deformity and the patients most likely to be helped by calf muscle lengthening procedures.

## Methods

### Patient collective

This study included a select group of 40 patients (80 feet) with clinically diagnosed CMT disease and associated cavovarus foot deformity. Patients had prior clinical and neurological evaluations which included family history, electromyography and genetic testing before referral to our specialty hospital. Radiographic evaluations had been done prior to or upon admission to our hospital. Of this cohort, 19 were male patients, and 21 female patients. Their average age was 33.6 ± 14.6 years (range 13.5–65.5 years).

The inclusion criteria for the study were: a confirmed diagnosis of CMT with cavovarus deformity and the ability to walk without any assistive device. Patients who had prior surgery to the lower extremities, current ulcerations on the feet, or presented with gait disturbances due to concomitant lower extremity pathology such as hip dysplasia, were excluded from the study.

In addition, 26 feet of 13 normal asymptomatic and healthy volunteers (mean age 43.9 ± 10.8 years) (range 24.7–65.0 years) were evaluated for comparison. Of these, 6 were women and 7 were men.

The local ethics committee (Ethikkommission der Medizinischen Fakultät Heidelberg) approved this study (S-458/2010). No external source of funding was used for this investigation.

### Instrumented gait analysis

**Fig. 1:**
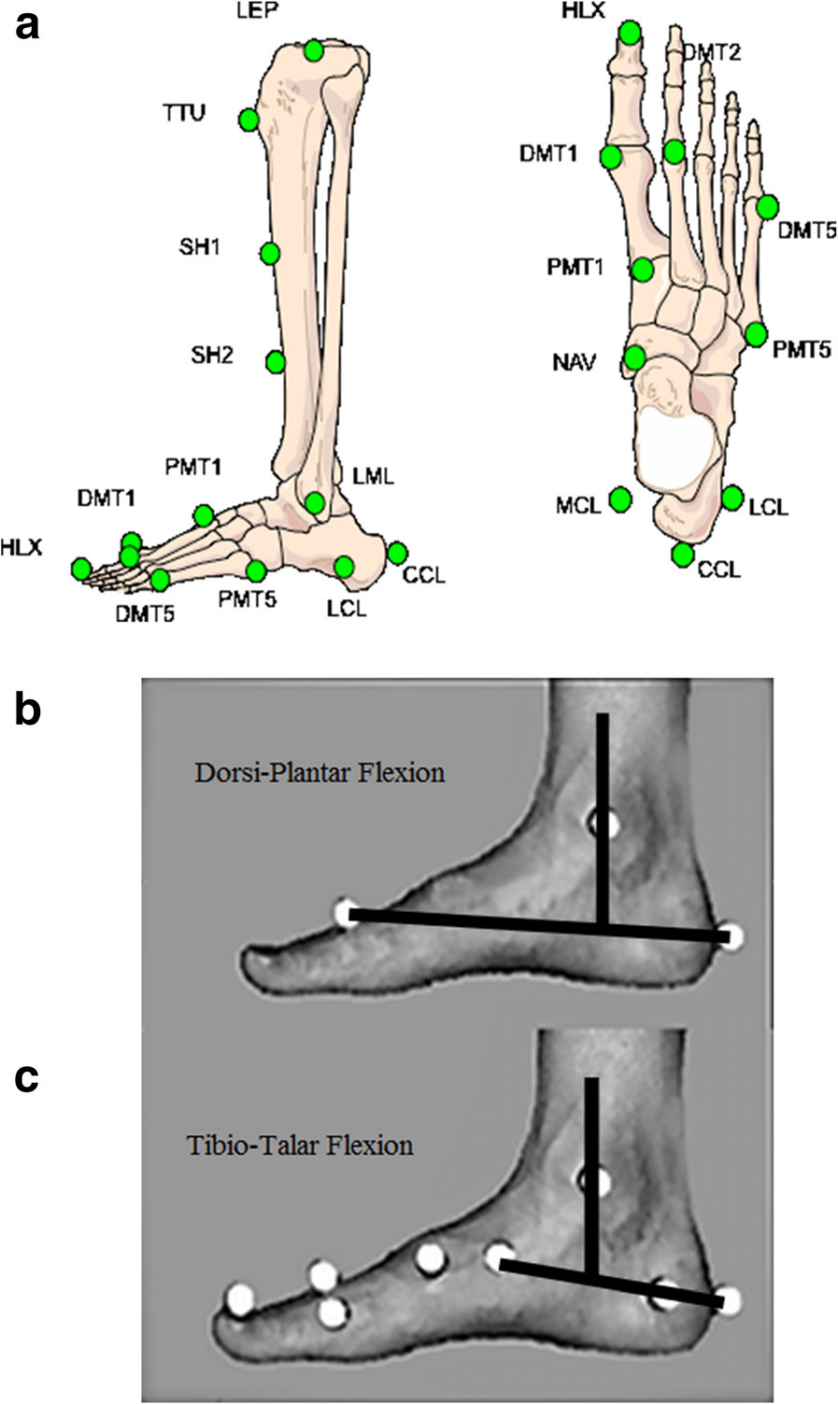
Depiction of the Heidelberg Foot Measurement Method (HFMM) marker placement according to Simon et al. [15] (modified from [12] and with approval from publisher). **a** Placement of markers in the lateral and medial epicondyles (LEP and MEP [not shown]), tibial tuberosity (TTU), two points on the medial side of the shin (SH1 and SH2), lateral and medial malleoli (LML and MML [not shown]), lateral, dorsal, and medial aspects of the calcaneus (LCL, CCL, and MCL), navicular (NAV), proximal and distal ends of the first metatarsal (P1MT and D1MT), hallux (HLX), distal end of the second metatarsal (D2MT), and distal and proximal ends of the fifth metatarsal (D5MT and P5MT). (see Simon et al. [15]). **b** Dorsiplantarflexion (flexion between the tibia and the medial longitudinal foot axis) is determined by the line between the calcaneus and the distal end of the first metatarsal (D1MT in Fig. 1a). Positive values = dorsiflexion, negative values = plantar flexion. This parameter describes the sagittal motion between the whole foot and the tibia (and is consequently influenced by the severity of the cavus deformity). **c** Tibiotalar flexion (flexion between the tibia and the talus, represented by the motion of the calcaneus and navicular) is calculated as the rotation around the malleolar line. Positive values = dorsiflexion, negative values = plantar flexion. This parameter evaluates ankle function independent of the midfoot and forefoot

All patients underwent instrumented gait analysis to further examine their respective kinematics before any operative treatment was performed. All subjects were examined using a standardized protocol. After clinical evaluation, a three-dimensional conventional Plug-in-Gait analysis utilizing a motion capture system (Vicon®, Oxford Metrics, UK) was done on all patients and controls. Skin mounted markers were applied on patients' predetermined bony landmarks according to a standardized protocol; the kinematics were calculated using a standard software procedure [19]. For the validated HFMM method [15], 17 reflective markers were positioned on well-defined bony landmarks on the lower leg and foot (see [15]) (Fig. 1a). To standardize the placement of markers at the calcaneus, a heel alignment device (HAD) was used. Markers were placed at the calcaneus and at the navicular bone for specific evaluation of the sagittal hindfoot motion (TTDF) relative to the tibia (Fig. 1a and c). Although TTDF does not show the motion of the talus directly, the motion in the talonavicular joint and subtalar joint in the sagittal plane was assumed to be very small relative to that of the ankle joint. For reasons of consistency with prior literature [15], we decided to continue to refer to the parameter as tibiotalar dorsiflexion. For evaluation of DPF, markers at the heads of the first, second and fifth metatarsal bones and the tibial markers were used [15] (Fig. 1a and b).

After marker placement, patients and controls were asked to walk barefoot along a 7 m walkway. A minimum of 10 valid strides were chosen to evaluate the marker trajectories. Kinematics were calculated using a customized Matlab® based software package, and the mean and standard deviations of these readings were obtained.

### Data analysis

To determine if a true hindfoot equinus deformity was present, three different parameters were chosen from both the conventional Plug-in-Gait analysis and the HFMM: from the conventional Plug-in-Gait analysis the DPF was chosen, which is a conventional parameter that describes the sagittal motion between the entire foot and tibia and is influenced by the extent of the cavus deformity. From the HFMM TTDF was chosen which describes the hindfoot motion relative to the tibia in the sagittal plane and MAA was chosen which quantitavely describes the longitudinal medial arch (degree of cavus). We determined the maximum TTDF as well as the maximum DPF and MAA in stance for each foot. The mean and standard deviations were calculated for these 80 feet. These values were then compared to those of the normal volunteer control group. CMT involved feet were classified as having increased TTDF (>1 SD above average of control group), normal TTDF (within 1 SD of average of control group) and decreased TTDF (>1 SD below average of control group) (Table 1).

**Table 1:** Maximum tibio-talar dorsiflexion (TTDF), dorsi-plantar flexion (DPF) and medial arch angle (MAA) in 26 normal feet and 80 feet of patients with CMT

	Tibio-talar dorsiflexion (°)	Dorsi-plantar flexion (°)	Medial arch angle (°)
	Mean (+/- 1 SD)	Mean (+/- 1 SD)	Mean (+/- 1 SD)
	(range)	(range)	(range)
Normal control (*N* = 26)	10.1 (+/− 3.0)	14.2 (+/− 4.1)	128.2 (+/− 5.6)
	(3.6 to 14.9)	(5.8 to 22.0)	(113.3 to 136.8)
Patients with CMT and normal TTDF (*N* = 31)	10.4 (+/− 1.8)	16.8 (+/− 7.0)	112.9 (+/− 12.6)
	(7.6 to 12.9)	((−7.3) to 38.5)	(78.5 to 133.3)
Patients with CMT and increased TTDF (*N* = 31)	16.1 (+/− 1.9)	20.5 (+/− 4.8)	111.6 (+/− 10.3)
	(13.6 to 21.1)	(1.8 to 30.9)	(90.3 to 129.1)
Patients with CMT and decreased TTDF (*N* = 18)	3.4 (+/− 3.0)	8.9 (+/− 6.0)	106.5 (+/− 16.2)
	((−2.4) to 7.1)	((−5.8) to 20.9)	(80.2 to 137.4)

### Statistical analysis

Basic statistical tests were used for data analysis. The data were evaluated descriptively using the arithmetic mean, standard deviation (SD), and minimum and maximum values. We created frequency and contingency tables, tested for normal distribution and calculated the Pearson's correlation coefficient and ANOVA to evaluate the respective groups. A *p*-value of ≤ 0.05 was considered significant. Statistical evaluation was performed using the analytical software IBM SPSS Statistics for Windows.

## Results

Table 1 shows the relationship between tibiotalar dorsiflexion (TTDF), dorsi-plantar flexion (DPF), and medial arch angle (MAA) in 26 normal feet (control population - NORM), and 80 feet of patients with CMT and cavovarus deformity. The Table shows that the range of motion in dorsi-plantar flexion was consistently greater than in tibio-talar dorsiflexion in both the normal controls and the feet with cavovarus deformity. Also the Table shows that the cavus component in the patient cohort was associated with a decreased MAA in the affected feet. Of particular note, only 18 (22.5 %) of the CMT affected feet showed decreased TTDF, 31 patient feet showed normal values (38.75 %) and 31 showed increased values (38.75 %) compared to the control group of normal feet.

The Pearson correlation between the TTDF and DPF was calculated for all 80 feet in addition to both the right and left feet separately. This correlation showed an *R* = 0.765 (*p* < 0.001) (Left side: *R* = 0.791, Right side: *R* = 0.770) in cases of decreased TTDF with HFMM and DPF with conventional Plug-in-Gait analysis and a very weak correlation in cases of normal TTDF (*R* = - 0.118) (Left side: *R* = 0.230, Right side: *R* = −0.397) and increased TTDF (*R* = 0.078) (Left side: *R* = 0.185, Right side: *R* = 0.070). Also, in cases with decreased TTDF there was a weak to moderate correlation with the MAA (*R* = 0.335) (Left side: *R* = 0.249, Right side: *R* = 0.387) but no correlation between MAA and DPF (*R* = 0.023) (Left side: *R* = −0.089, Right side: *R* = 0.132).

**Fig. 2:**
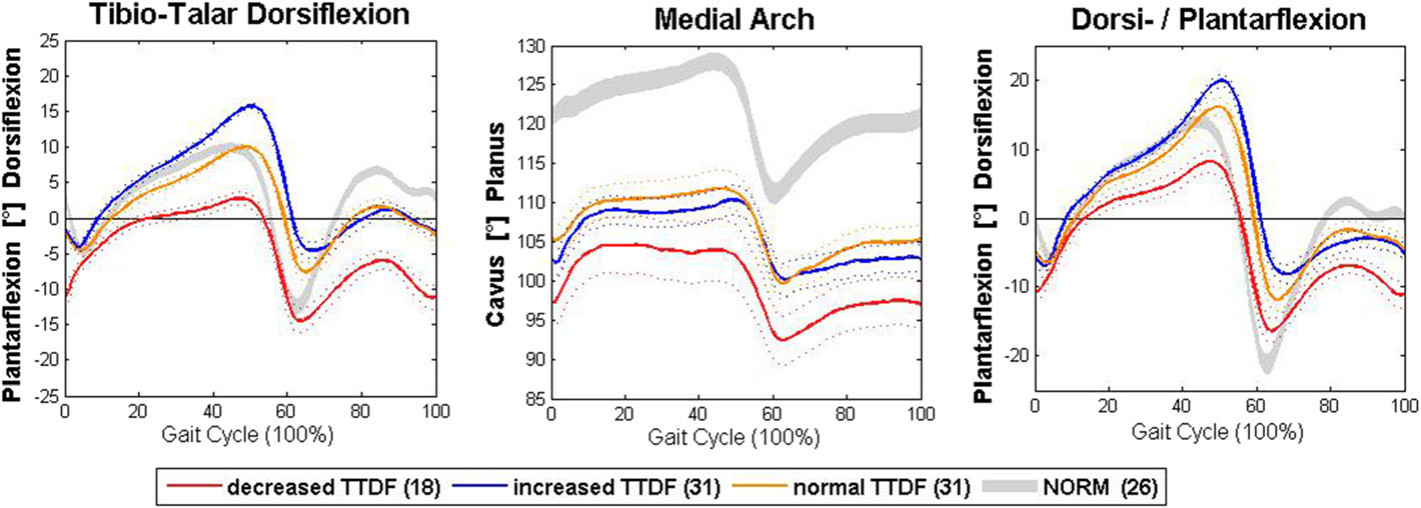
Results of the three-dimensional foot analysis (tibiotalar dorsiflexion, dorsiplantar flexion, and medial arch angle) are shown during the stance and swing phases of the gait cycle as averaged curves of the feet. Red depicts the decreased tibiotalar dorsiflexion group, blue the increased tibiotalar dorsiflexion group, and green the group with tibiotalar dorsiflexion within 1 standard deviation (SD) of the norm (grey)). Each solid line represents the mean, while the dotted lines represent 1 SD above or below the mean. The range for twenty-six normal feet is represented by the grey shading

In Fig. 2, all phases of the gait cycle during TTDF motion, DPF motion and medial arch angle changes are represented graphically for the 3 groups of patients with normal, increased and decreased maximal tibio-talar dorsiflexion (TTDF) values. In patients with decreased maximal TTDF values, all 3 curves fell below those of the normal control group (NORM). Patients with normal TTDF values also displayed decreased DPF except during the pre-swing phase. The patients with decreased TTDF values also displayed a lower MAA (indicating increased cavus) compared to patients with normal or increased TTDF values (Table 1).

## Discussion

The goal of this study was to more accurately and specifically define the kinematics of CMT patients with CMT disease and cavovarus deformity by studying specific sagittal hindfoot kinematics in addition to conventional foot motion, in order to identify patients with true hindfoot equinus deformity and tailor their treatment more appropriately.

The previously validated 3 dimensional HFMM [15] allowed us to separate, define and measure hindfoot motion (TTDF (Fig. 1c)) independent of the overall motion of the forefoot and the tibia (DPF) (Fig. 1b). We were able to show that in our patient cohort with CMT, only 22.5 % of feet studied had decreased TTDF in stance phase consistent with shortening of the calf muscles and resultant hindfoot equinus deformity. Conventional Plug-in-Gait analysis [6, 14, 20] did not separate the motion of the hindfoot from that of the mid- or forefoot and therefore did not identify the hindfoot equinus component of a cavus deformity.

Our study documents the advantage of utilizing the HFMM in these cases to identify segmental deformities, and unlike conventional Plug-in-Gait analysis, also defines distinct deformity groups with varying degrees of equinus deformity. In addition, patients with decreased TTDF indicating presence of hindfoot equinus identified by the HFMM, correlate positively with patients with decreased DPF identified by conventional Plug-in-Gait analysis; however, it must be noted that the converse does not apply - i.e., not all cases of decreased DPF on conventional Plug-in-Gait analysis have decreased TTDF with HFMM. Severe cavus deformities can result in decreased DPF with conventional Plug-in-Gait analysis and have only a mild to no equinus deformity when evaluated with HFMM.

Conventional Plug-in-Gait analysis is limited in its analytic scope and does not indicate if a decreased DPF is a result of a cavus or a hindfoot equinus deformity. Consequently, conventional Plug-in-Gait analysis is limited when used as the only gait analysis tool for making treatment related decisions. Our study indicates that if conventional Plug-in-Gait analysis alone is used in these patients, there is the risk of diagnosing a spuriously increased incidence of equinus foot deformity resulting in unnecessary calf muscle or Achilles tendon lengthening procedures in some patients who already suffer from weakened muscles and impaired muscular balance. These patients are placed at an increased risk of developing a worsened or calcaneal gait.

Prior studies using conventional Plug-in-Gait analysis [6, 14, 20–22] have shown that patients with CMT do not display uniform gait analysis findings. Don et al. [21] described 2 distinct gait patterns that they ascribed to the degree of foot drop and plantar flexion failure, attributed to severe weakness of both the ankle dorsi-flexor and plantar flexor muscles. They also pointed out that the excessively plantar flexed ankle at initial contact forces patients with CMT to perform a large dorsi-flexion movement during the stance phase in order to maintain an adequate step length. This was noted in 31 of the 80 feet of our patients.

Ounpuu et al. [20] defined 3 subgroups based on peak DPF at the end of stance phase of the gait cycle: patients with increased, normal or decreased DPF. They felt that the group with decreased dorsi-plantar flexion (DPF) had associated contracture of the plantar flexor muscles. Our data showed that these patients with severe cavus may demonstrate decreased DPF with conventional Plug-in-Gait analysis but have only a mild or absent equinus deformity when evaluated with HFMM (based on a normal or increased TTDF). In these cases a calf muscle lengthening procedure could be deleterious and result in weakened plantar flexors or calcaneal gait.

Prior methods for evaluation of hindfoot flexibility have been described. Already in 1977 Coleman et al. [23] described one method, which was later modified to a radiographic test by Azmaipairashvili et al. [24]. During weight bearing the heel and lateral foot are placed on a one inch wooden block,and simultaneously the first to third metatarsals are allowed to fall passively into pronation. If during this maneuver the hindfoot corrects passively from varus to valgus position, the flexibility is preserved. However, this test does not specifically address the equinus deformity. Another radiographic test was described by Aktas et al. [25], who analyzed 26 patients with CMT and pes cavus deformity by obtaining lateral weight bearing radiographs of the foot. They found that the hindfoot in these patients is generally dorsiflexed as shown by the tibio-talar and tibiocalcaneal angle and is not in equinus position. According to these authors the apparent equinus deformity is secondary to plantar flexion of the forefoot on the midfoot and in fact represents a cavus deformity. However, all these x-ray studies are based on static images, taken while standing, and do not evaluate the hindfoot and forefoot motion.

In patients with a true equinus deformity involving the hindfoot, Achilles tendon or calf muscle lengthening is indicated. However, Achilles tendon lengthening can lead to weakening of the flexor muscles [26–28] and possible permanent impairment, even in patients without peripheral neuropathy [29]. Consequently, in patients with pre-existing weakness of the flexor muscles, this procedure should only be performed in very carefully selected cases in order to avoid the development of a calcaneal gait. Conventional Plug-in-Gait analysis provides insufficient information about the cavovarus deformity and may lead to an inaccurate clinical assessment. A detailed foot segment analysis is essential to assess the complex cavovarus deformity and can provide substantial necessary information to aid further treatment.

This study is limited in part by the lack of an exact calibration method for determining the neutral position of the calcaneus. We used the heel alignment device to position the marker on the calcaneus. However, due to the variability of the heel fat pad, and subcutaneous fat thickness in different subjects, an exact anatomic placement was not certain. In future studies, radiographic correlation of a placed marker could allow improved assessment of the actual orientation of the hindfoot, and consequently be used to calculate offset angles. In addition, by selecting only patients with CMT and cavovarus deformity, we are excluding a large number of patients who did not meet the inclusion criteria. We also excluded patients who were unable to participate in the gait analysis (unable to walk unassisted), had prior surgery to the lower extremities, or gait disturbances secondary to lower leg pathology. This selection process may have excluded patients with the most severe forms of the disorder, so that our cohort was possibly skewed towards milder cases. However, the purpose of this study was to specifically identify the presence of hindfoot equinus deformity irrespective of subtype or severity of the disease in order to identify the most appropriate corrective surgery for this specific deformity.

## Conclusions

In summary, conventional Plug-in-Gait analysis is limited in its ability to delineate complex foot deformities and their effect on hindfoot motion. A segmental evaluation of forefoot and hindfoot motion as done with the HFMM provides important adjunctive information for planning the most appropriate and effective surgical correction of cavovarus deformity in patients with CMT disease. Although there is no specific marker on the talus and TTDF does not show the motion of the talus directly, we believe that the talonavicular joint and subtalar joint contribute minimally to the tibio-talar motion. Our data show that in our collective of patients with CMT, cavovarus foot deformity and associated foot drop, intrinsic calf muscle shortening was present in only a minority of the total cohort, and was present in only 22.5 % of our patient cohort as the cause of the equinus deformity. Achilles tendon or calf muscle lengthening should only be considered as an addition to the surgical correction of the cavovarus deformity in these few cases. In contrast to the HFMM, conventional Plug-in-Gait analysis may indicate a spuriously increased incidence of hindfoot equinus deformity, and was of limited value in assessing our patient collective. In patients who already suffer from pre-existing weakness of the muscles of the foot and lower leg secondary to their underlying disease, unnecessary calf muscle lengthening surgery may lead to severe calcaneal gait and should be avoided. In these patients only correction of the cavovarus deformity is indicated.
